# *N*-(2-{[5-Bromo-2-(piperidin-1-yl)pyrimidin-4-yl]sulfan­yl}-4-meth­oxy­phen­yl)-2,4,6-trimethyl­benzene­sulfonamide

**DOI:** 10.1107/S1600536812036185

**Published:** 2012-08-25

**Authors:** Mohan Kumar, L. Mallesha, M. A. Sridhar, Kamini Kapoor, Vivek K. Gupta, Rajni Kant

**Affiliations:** a Department of Studies in Physics, Manasagangotri, University of Mysore, Mysore 570 006, India; b PG Department of Studies in Chemistry, JSS College of Arts, Commerce and Science, Ooty Road, Mysore 570 025, India; c X-ray Crystallography Laboratory, Post-Graduate Department of Physics & Electronics, University of Jammu, Jammu Tawi 180 006, India

## Abstract

In the title compound, C_25_H_29_BrN_4_O_3_S_2_, the benzene rings bridged by the sulfonamide group are tilted relative to each other by 63.9 (1)° and the dihedral angle between the sulfur-bridged pyrimidine and benzene rings is 64.9 (1)°. The mol­ecular conformation is stabilized by a weak intra­molecular π–π stacking inter­action between the pyrimidine and the 2,4,6-trimethyl­benzene rings [centroid–centroid distance = 3.766 (2) Å]. The piperidine ring adopts a chair conformation. In the crystal, mol­ecules are linked into inversion dimers by pairs of N—H⋯O hydrogen bonds and these dimers are further linked by C—H⋯O hydrogen bonds into chains propagating along [010].

## Related literature

For the crystal structures of related sulfonamides, see: Rodrigues *et al.* (2011 ▶); Akkurt *et al.* (2011 ▶); Kant *et al.* (2012 ▶).
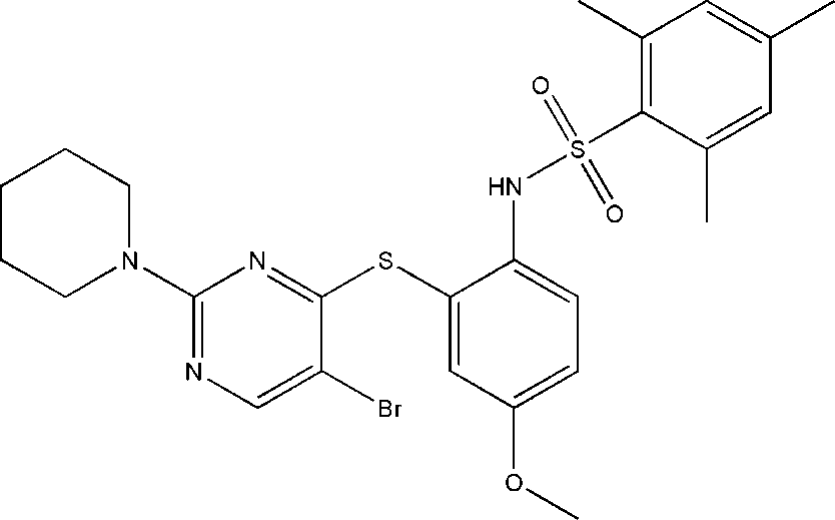


## Experimental

### 

#### Crystal data

C_25_H_29_BrN_4_O_3_S_2_
*M* *_r_* = 577.55
Monoclinic, 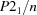

*a* = 9.3334 (5) Å
*b* = 10.3635 (4) Å
*c* = 27.8258 (11) Å
β = 92.924 (4)°
*V* = 2688.0 (2) Å^3^
*Z* = 4
Mo *K*α radiation
μ = 1.72 mm^−1^
*T* = 293 K
0.3 × 0.2 × 0.2 mm

#### Data collection

Oxford Diffraction Xcalibur Sapphire3 diffractometer
Absorption correction: multi-scan (*CrysAlis PRO*; Oxford Diffraction, 2010 ▶) *T*_min_ = 0.649, *T*_max_ = 1.000
21429 measured reflections
5266 independent reflections
3580 reflections with *I* > 2σ(*I*)
*R*_int_ = 0.043

#### Refinement

*R*[*F* ^2^ > 2σ(*F* ^2^)] = 0.054
*wR*(*F* ^2^) = 0.123
*S* = 1.06
5266 reflections
320 parameters
H-atom parameters constrained
Δρ_max_ = 0.35 e Å^−3^
Δρ_min_ = −0.43 e Å^−3^

### 

Data collection: *CrysAlis PRO* (Oxford Diffraction, 2010 ▶); cell refinement: *CrysAlis PRO*; data reduction: *CrysAlis PRO*; program(s) used to solve structure: *SHELXS97* (Sheldrick, 2008 ▶); program(s) used to refine structure: *SHELXL97* (Sheldrick, 2008 ▶); molecular graphics: *ORTEP-3* (Farrugia, 1997 ▶); software used to prepare material for publication: *PLATON* (Spek, 2009 ▶).

## Supplementary Material

Crystal structure: contains datablock(s) I, global. DOI: 10.1107/S1600536812036185/hb6940sup1.cif

Structure factors: contains datablock(s) I. DOI: 10.1107/S1600536812036185/hb6940Isup2.hkl

Supplementary material file. DOI: 10.1107/S1600536812036185/hb6940Isup3.cml

Additional supplementary materials:  crystallographic information; 3D view; checkCIF report

## Figures and Tables

**Table 1:** Hydrogen-bond geometry (Å, °)

*D*—H⋯*A*	*D*—H	H⋯*A*	*D*⋯*A*	*D*—H⋯*A*
N1—H1⋯O2^i^	0.86	2.03	2.880 (5)	172
C8—H8*A*⋯O2^ii^	0.96	2.48	3.242 (5)	136
C11—H11⋯O1^iii^	0.93	2.50	3.387 (6)	159

Symmetry codes: (i) 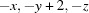; (ii) 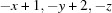; (iii) 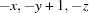.

**Fig. 1.**
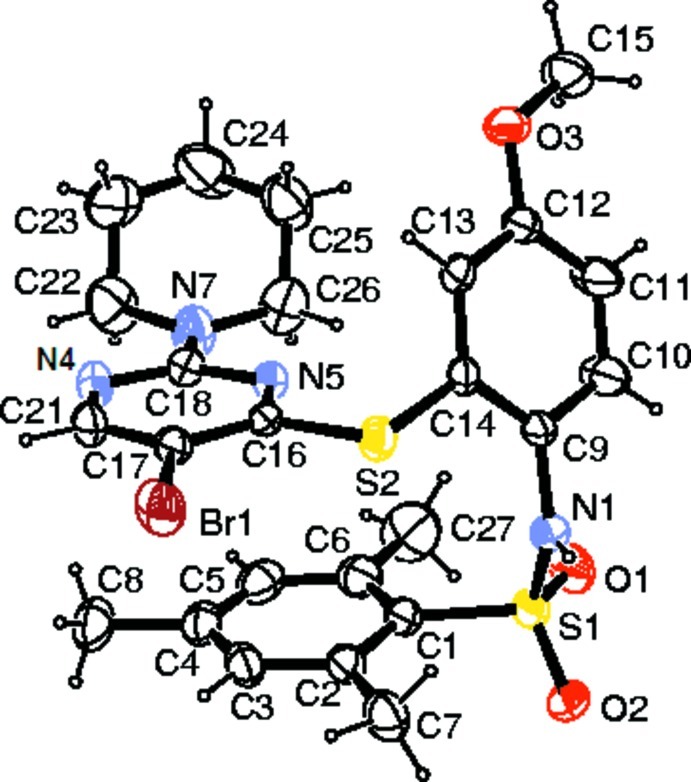
View of the molecule with displacement ellipsoids drawn at the 40% probability level.

**Fig. 2.**
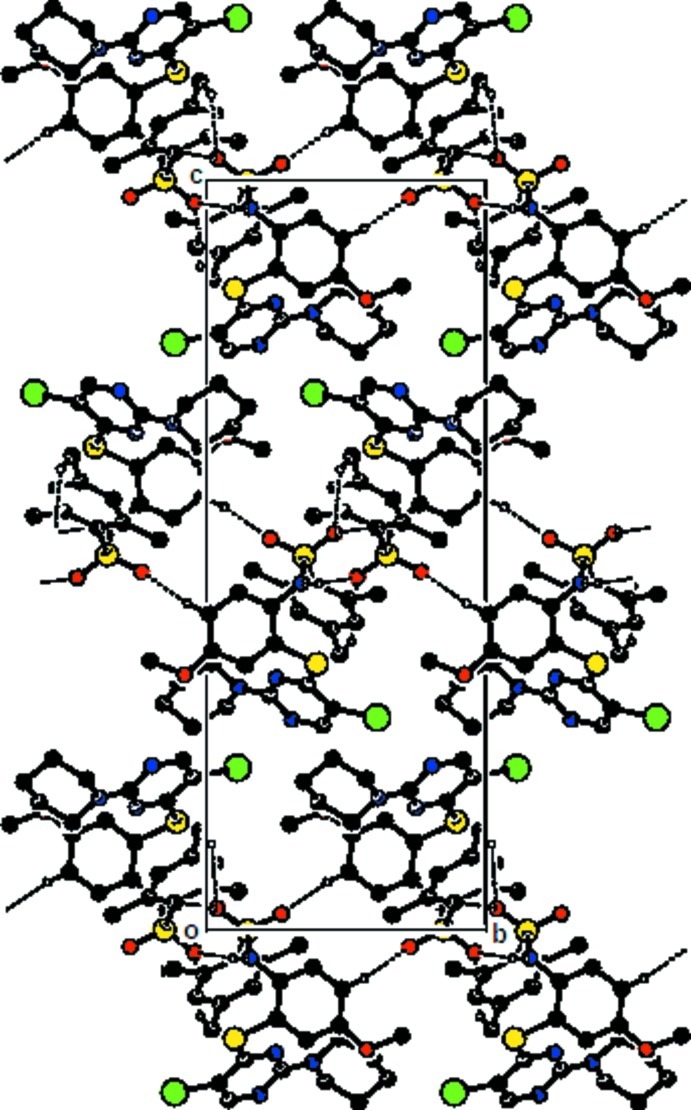
A molecular packing view of the title compound down the a axis, showing intermolecular interactions. For clarity, hydrogen atoms which are not involved in hydrogen bonding have been omitted.

## supplementary crystallographic information

### Comment

Bond lengths and angles in the title compound (Fig. 1) are comparable with those in similar crystal structures (Rodrigues *et al.*, 2011; Akkurt *et al.*, 2011; Kant *et al.*, 2012). The piperidine ring is exhibiting a chair conformation. The two benzene rings (C1—C6/C9—C14) are tilted relative to each other by 63.9 (1)° and the dihedral angle between the sulfur bridged pyrimidine and benzene rings is 64.9 (1)°. The molecular conformation is stabilized by a weak intramolecular stacking interaction between the pyrimidine and the 2,4,6 -trimethyl benzene rings [centroid–centroid distance = 3.766 (2) Å, interplanar spacing = 3.507 Å, and centroid shift = 1.37 Å]. In the crystal, molecules are linked into dimers by pairs of N1—H1···O2 hydrogen bonds and these dimers are further linked by C—H···O hydrogen bonds into chains along [010](Fig.2).

### Experimental

The reaction of *N*-[2-(5-bromo-2-chloro-pyrimidin-4-ylsulfanyl)-4-methoxy-phenyl]-2,4,6-trimethyl -benzenesulfonamide(5.29 g, 0.01 mol) with piperidine (0.86 g, 0.01) were carried out in the presence of triethylamine and the reaction mixture was allowed to stir at room temperature for 6–7 h in dry dichloromethane. The progress of the reaction was monitored by TLC. Upon completion, the solvent was removed under reduced pressure and residue was extracted with ethyl acetate. The compound was purified by successive recrystallization from methanol (yield 82%, m.p. 460–462 K).

### Refinement

All H atoms were positioned geometrically and were treated as riding on their parent C/N atoms, with C—H distances of 0.93–0.97 Å and N—H distance of 0.86 with *U*_iso_(H) = 1.2*U*_eq_(C) or 1.5*U*_eq_(methyl C).

### Crystal data

**Table:**

C_25_H_29_BrN_4_O_3_S_2_	*F*(000) = 1192
*M**_r_* = 577.55	*D*_x_ = 1.427 Mg m^−^^3^
Monoclinic, *P*2_1_/*n*	Mo *K*α radiation, λ = 0.71073 Å
Hall symbol: -P 2yn	Cell parameters from 6963 reflections
*a* = 9.3334 (5) Å	θ = 3.5–29.0°
*b* = 10.3635 (4) Å	µ = 1.72 mm^−^^1^
*c* = 27.8258 (11) Å	*T* = 293 K
β = 92.924 (4)°	Block, white
*V* = 2688.0 (2) Å^3^	0.3 × 0.2 × 0.2 mm
*Z* = 4	

### Data collection

**Table:**

Oxford Diffraction Xcalibur Sapphire3 diffractometer	5266 independent reflections
Radiation source: fine-focus sealed tube	3580 reflections with *I* > 2σ(*I*)
Graphite monochromator	*R*_int_ = 0.043
Detector resolution: 16.1049 pixels mm^-1^	θ_max_ = 26.0°, θ_min_ = 3.5°
ω scan	*h* = −11→11
Absorption correction: multi-scan (*CrysAlis PRO*; Oxford Diffraction, 2010)	*k* = −12→12
*T*_min_ = 0.649, *T*_max_ = 1.000	*l* = −34→34
21429 measured reflections	

### Refinement

**Table:**

Refinement on *F*^2^	Primary atom site location: structure-invariant direct methods
Least-squares matrix: full	Secondary atom site location: difference Fourier map
*R*[*F*^2^ > 2σ(*F*^2^)] = 0.054	Hydrogen site location: inferred from neighbouring sites
*wR*(*F*^2^) = 0.123	H-atom parameters constrained
*S* = 1.06	*w* = 1/[σ^2^(*F*_o_^2^) + (0.0365*P*)^2^ + 2.6423*P*] where *P* = (*F*_o_^2^ + 2*F*_c_^2^)/3
5266 reflections	(Δ/σ)_max_ = 0.001
320 parameters	Δρ_max_ = 0.35 e Å^−^^3^
0 restraints	Δρ_min_ = −0.43 e Å^−^^3^

### Special details

**Table:**

Experimental. *CrysAlis PRO*, Oxford Diffraction Ltd., Version 1.171.34.40 (release 27–08-2010 CrysAlis171. NET) (compiled Aug 27 2010,11:50:40) Empirical absorption correction using spherical harmonics, implemented in SCALE3 ABSPACK scaling algorithm.
Geometry. All e.s.d.'s (except the e.s.d. in the dihedral angle between two l.s. planes) are estimated using the full covariance matrix. The cell e.s.d.'s are taken into account individually in the estimation of e.s.d.'s in distances, angles and torsion angles; correlations between e.s.d.'s in cell parameters are only used when they are defined by crystal symmetry. An approximate (isotropic) treatment of cell e.s.d.'s is used for estimating e.s.d.'s involving l.s. planes.
Refinement. Refinement of *F*^2^ against ALL reflections. The weighted *R*-factor *wR* and goodness of fit *S* are based on *F*^2^, conventional *R*-factors *R* are based on *F*, with *F* set to zero for negative *F*^2^. The threshold expression of *F*^2^ > σ(*F*^2^) is used only for calculating *R*-factors(gt) *etc*. and is not relevant to the choice of reflections for refinement. *R*-factors based on *F*^2^ are statistically about twice as large as those based on *F*, and *R*- factors based on ALL data will be even larger.

### Fractional atomic coordinates and isotropic or equivalent isotropic displacement parameters (Å^2^)

**Table:**

	*x*	*y*	*z*	*U*_iso_*/*U*_eq_	
Br1	0.24625 (5)	1.11199 (4)	0.216359 (17)	0.07602 (19)	
S1	0.19179 (12)	0.85193 (11)	0.00058 (3)	0.0626 (3)	
S2	0.10025 (10)	0.89654 (9)	0.14471 (3)	0.0513 (3)	
O1	0.2090 (4)	0.7302 (3)	−0.02194 (10)	0.0849 (10)	
O2	0.1541 (3)	0.9599 (3)	−0.02939 (9)	0.0725 (9)	
O3	−0.0197 (3)	0.4244 (3)	0.15889 (9)	0.0700 (8)	
N1	0.0612 (4)	0.8411 (3)	0.03700 (10)	0.0628 (9)	
H1	0.0005	0.9035	0.0374	0.075*	
N4	0.5272 (3)	0.8077 (3)	0.21918 (12)	0.0604 (9)	
N5	0.3329 (3)	0.7523 (3)	0.16362 (10)	0.0453 (7)	
N7	0.5265 (4)	0.6181 (3)	0.17624 (14)	0.0733 (10)	
C1	0.3494 (4)	0.8878 (4)	0.03643 (12)	0.0523 (9)	
C2	0.3591 (4)	1.0096 (4)	0.05852 (13)	0.0518 (9)	
C3	0.4791 (4)	1.0346 (4)	0.08927 (14)	0.0612 (11)	
H3	0.4856	1.1145	0.1045	0.073*	
C4	0.5867 (5)	0.9480 (5)	0.09806 (14)	0.0646 (12)	
C5	0.5758 (5)	0.8317 (5)	0.07489 (15)	0.0699 (12)	
H5	0.6498	0.7725	0.0801	0.084*	
C6	0.4601 (5)	0.7970 (4)	0.04384 (15)	0.0652 (11)	
C7	0.2507 (5)	1.1154 (4)	0.05177 (17)	0.0712 (12)	
H7A	0.2515	1.1474	0.0194	0.107*	
H7B	0.2740	1.1842	0.0739	0.107*	
H7C	0.1571	1.0825	0.0576	0.107*	
C8	0.7134 (5)	0.9787 (6)	0.13240 (16)	0.0922 (17)	
H8A	0.7873	1.0196	0.1151	0.138*	
H8B	0.7499	0.9002	0.1467	0.138*	
H8C	0.6832	1.0356	0.1572	0.138*	
C9	0.0417 (4)	0.7345 (4)	0.06810 (12)	0.0555 (10)	
C10	−0.0028 (6)	0.6177 (5)	0.04949 (15)	0.0934 (18)	
H10	−0.0200	0.6100	0.0164	0.112*	
C11	−0.0228 (6)	0.5119 (5)	0.07801 (15)	0.0879 (17)	
H11	−0.0506	0.4335	0.0642	0.106*	
C12	−0.0016 (4)	0.5222 (4)	0.12717 (13)	0.0549 (10)	
C13	0.0332 (4)	0.6404 (4)	0.14644 (12)	0.0500 (9)	
H13	0.0404	0.6497	0.1797	0.060*	
C14	0.0579 (4)	0.7461 (4)	0.11776 (12)	0.0449 (8)	
C15	−0.0514 (6)	0.2991 (4)	0.14117 (17)	0.0791 (14)	
H15A	−0.1376	0.3018	0.1209	0.119*	
H15B	−0.0645	0.2418	0.1677	0.119*	
H15C	0.0265	0.2689	0.1229	0.119*	
C16	0.2696 (4)	0.8624 (3)	0.17246 (11)	0.0406 (8)	
C17	0.3325 (4)	0.9525 (3)	0.20396 (12)	0.0466 (9)	
C18	0.4616 (4)	0.7291 (4)	0.18658 (13)	0.0504 (9)	
C21	0.4617 (4)	0.9181 (4)	0.22662 (13)	0.0580 (10)	
H21	0.5051	0.9760	0.2484	0.070*	
C22	0.6581 (5)	0.5732 (5)	0.2017 (2)	0.0938 (16)	
H22A	0.6940	0.6396	0.2237	0.113*	
H22B	0.7306	0.5566	0.1787	0.113*	
C23	0.6303 (6)	0.4531 (6)	0.2290 (2)	0.110 (2)	
H23A	0.5689	0.4732	0.2551	0.131*	
H23B	0.7204	0.4206	0.2431	0.131*	
C24	0.5602 (7)	0.3496 (6)	0.1980 (3)	0.124 (2)	
H24A	0.5336	0.2776	0.2179	0.148*	
H24B	0.6275	0.3183	0.1752	0.148*	
C25	0.4283 (6)	0.4025 (5)	0.1711 (2)	0.1031 (19)	
H25A	0.3898	0.3377	0.1488	0.124*	
H25B	0.3556	0.4214	0.1938	0.124*	
C26	0.4612 (6)	0.5222 (5)	0.14396 (18)	0.0857 (15)	
H26A	0.5261	0.5019	0.1189	0.103*	
H26B	0.3735	0.5567	0.1288	0.103*	
C27	0.4663 (7)	0.6661 (5)	0.0203 (2)	0.113 (2)	
H27A	0.5549	0.6244	0.0300	0.169*	
H27B	0.4606	0.6762	−0.0141	0.169*	
H27C	0.3873	0.6144	0.0299	0.169*	

### Atomic displacement parameters (Å^2^)

**Table:**

	*U*^11^	*U*^22^	*U*^33^	*U*^12^	*U*^13^	*U*^23^
Br1	0.0919 (4)	0.0579 (3)	0.0777 (3)	−0.0032 (2)	−0.0005 (3)	−0.0240 (2)
S1	0.0804 (8)	0.0720 (7)	0.0346 (5)	−0.0331 (6)	−0.0047 (5)	0.0008 (5)
S2	0.0549 (6)	0.0503 (5)	0.0476 (5)	−0.0009 (5)	−0.0081 (4)	−0.0051 (4)
O1	0.117 (3)	0.085 (2)	0.0528 (17)	−0.039 (2)	−0.0007 (17)	−0.0228 (16)
O2	0.077 (2)	0.095 (2)	0.0442 (15)	−0.0272 (17)	−0.0060 (14)	0.0220 (15)
O3	0.103 (2)	0.0584 (17)	0.0485 (15)	−0.0251 (16)	0.0001 (15)	0.0052 (14)
N1	0.069 (2)	0.077 (2)	0.0421 (17)	−0.0288 (19)	−0.0059 (15)	0.0138 (17)
N4	0.055 (2)	0.067 (2)	0.058 (2)	−0.0087 (18)	−0.0134 (16)	−0.0050 (18)
N5	0.0479 (18)	0.0480 (18)	0.0394 (16)	−0.0064 (14)	−0.0029 (13)	−0.0011 (14)
N7	0.067 (2)	0.062 (2)	0.088 (3)	0.0127 (19)	−0.018 (2)	−0.008 (2)
C1	0.063 (2)	0.055 (2)	0.0388 (19)	−0.016 (2)	0.0010 (17)	−0.0008 (18)
C2	0.051 (2)	0.059 (2)	0.046 (2)	−0.013 (2)	0.0029 (17)	0.0005 (19)
C3	0.061 (3)	0.073 (3)	0.049 (2)	−0.024 (2)	0.003 (2)	−0.009 (2)
C4	0.052 (3)	0.099 (4)	0.043 (2)	−0.016 (3)	0.0038 (19)	0.008 (2)
C5	0.060 (3)	0.089 (3)	0.061 (3)	0.007 (3)	0.008 (2)	0.016 (3)
C6	0.080 (3)	0.062 (3)	0.054 (2)	−0.008 (2)	0.010 (2)	−0.003 (2)
C7	0.075 (3)	0.061 (3)	0.077 (3)	−0.009 (2)	−0.004 (2)	−0.013 (2)
C8	0.058 (3)	0.154 (5)	0.063 (3)	−0.027 (3)	−0.008 (2)	0.016 (3)
C9	0.067 (3)	0.063 (2)	0.0353 (19)	−0.032 (2)	−0.0038 (17)	0.0024 (18)
C10	0.156 (5)	0.090 (4)	0.034 (2)	−0.069 (4)	0.004 (3)	−0.008 (2)
C11	0.146 (5)	0.075 (3)	0.043 (2)	−0.061 (3)	0.013 (3)	−0.012 (2)
C12	0.067 (3)	0.057 (2)	0.041 (2)	−0.023 (2)	0.0038 (18)	0.0000 (19)
C13	0.054 (2)	0.064 (3)	0.0313 (18)	−0.0146 (19)	−0.0020 (16)	−0.0010 (18)
C14	0.043 (2)	0.055 (2)	0.0353 (18)	−0.0125 (17)	−0.0051 (15)	−0.0019 (17)
C15	0.102 (4)	0.052 (3)	0.083 (3)	−0.015 (3)	0.005 (3)	0.000 (2)
C16	0.048 (2)	0.045 (2)	0.0282 (16)	−0.0126 (17)	0.0002 (14)	0.0024 (15)
C17	0.055 (2)	0.048 (2)	0.0370 (19)	−0.0115 (18)	0.0042 (17)	−0.0042 (16)
C18	0.051 (2)	0.054 (2)	0.046 (2)	−0.0048 (19)	−0.0029 (18)	0.0033 (18)
C21	0.065 (3)	0.064 (3)	0.045 (2)	−0.019 (2)	−0.0068 (19)	−0.009 (2)
C22	0.058 (3)	0.093 (4)	0.129 (5)	0.014 (3)	−0.007 (3)	0.003 (4)
C23	0.064 (3)	0.132 (5)	0.131 (5)	0.015 (3)	−0.015 (3)	0.053 (4)
C24	0.085 (4)	0.083 (4)	0.200 (7)	−0.002 (3)	−0.019 (4)	0.042 (5)
C25	0.088 (4)	0.073 (3)	0.145 (5)	−0.006 (3)	−0.022 (4)	0.006 (4)
C26	0.099 (4)	0.072 (3)	0.085 (3)	0.023 (3)	−0.010 (3)	−0.016 (3)
C27	0.159 (6)	0.076 (4)	0.102 (4)	0.015 (4)	−0.002 (4)	−0.023 (3)

### Geometric parameters (Å, º)

**Table:**

Br1—C17	1.878 (4)	C8—H8C	0.9600
S1—O1	1.422 (3)	C9—C10	1.373 (5)
S1—O2	1.429 (3)	C9—C14	1.388 (5)
S1—N1	1.628 (3)	C10—C11	1.372 (6)
S1—C1	1.774 (4)	C10—H10	0.9300
S2—C16	1.760 (4)	C11—C12	1.376 (5)
S2—C14	1.766 (4)	C11—H11	0.9300
O3—C12	1.360 (4)	C12—C13	1.369 (5)
O3—C15	1.415 (5)	C13—C14	1.382 (5)
N1—C9	1.420 (5)	C13—H13	0.9300
N1—H1	0.8600	C15—H15A	0.9600
N4—C21	1.319 (5)	C15—H15B	0.9600
N4—C18	1.343 (5)	C15—H15C	0.9600
N5—C16	1.313 (4)	C16—C17	1.390 (4)
N5—C18	1.353 (4)	C17—C21	1.379 (5)
N7—C18	1.339 (5)	C21—H21	0.9300
N7—C26	1.452 (6)	C22—C23	1.488 (7)
N7—C22	1.461 (6)	C22—H22A	0.9700
C1—C2	1.405 (5)	C22—H22B	0.9700
C1—C6	1.405 (6)	C23—C24	1.505 (8)
C2—C3	1.398 (5)	C23—H23A	0.9700
C2—C7	1.497 (6)	C23—H23B	0.9700
C3—C4	1.360 (6)	C24—C25	1.511 (7)
C3—H3	0.9300	C24—H24A	0.9700
C4—C5	1.368 (6)	C24—H24B	0.9700
C4—C8	1.516 (6)	C25—C26	1.493 (7)
C5—C6	1.395 (6)	C25—H25A	0.9700
C5—H5	0.9300	C25—H25B	0.9700
C6—C27	1.509 (6)	C26—H26A	0.9700
C7—H7A	0.9600	C26—H26B	0.9700
C7—H7B	0.9600	C27—H27A	0.9600
C7—H7C	0.9600	C27—H27B	0.9600
C8—H8A	0.9600	C27—H27C	0.9600
C8—H8B	0.9600		
			
O1—S1—O2	117.88 (18)	C12—C13—H13	119.1
O1—S1—N1	108.56 (19)	C14—C13—H13	119.1
O2—S1—N1	104.34 (19)	C13—C14—C9	119.6 (3)
O1—S1—C1	108.9 (2)	C13—C14—S2	119.7 (3)
O2—S1—C1	109.66 (17)	C9—C14—S2	120.7 (3)
N1—S1—C1	106.87 (16)	O3—C15—H15A	109.5
C16—S2—C14	100.67 (17)	O3—C15—H15B	109.5
C12—O3—C15	119.2 (3)	H15A—C15—H15B	109.5
C9—N1—S1	123.8 (3)	O3—C15—H15C	109.5
C9—N1—H1	118.1	H15A—C15—H15C	109.5
S1—N1—H1	118.1	H15B—C15—H15C	109.5
C21—N4—C18	115.7 (3)	N5—C16—C17	121.5 (3)
C16—N5—C18	117.5 (3)	N5—C16—S2	119.7 (2)
C18—N7—C26	122.7 (4)	C17—C16—S2	118.8 (3)
C18—N7—C22	123.2 (4)	C21—C17—C16	116.4 (3)
C26—N7—C22	113.4 (4)	C21—C17—Br1	121.1 (3)
C2—C1—C6	120.5 (4)	C16—C17—Br1	122.4 (3)
C2—C1—S1	117.9 (3)	N7—C18—N4	118.1 (4)
C6—C1—S1	121.6 (3)	N7—C18—N5	116.9 (3)
C3—C2—C1	117.8 (4)	N4—C18—N5	125.1 (4)
C3—C2—C7	117.1 (4)	N4—C21—C17	123.7 (3)
C1—C2—C7	125.1 (3)	N4—C21—H21	118.2
C4—C3—C2	123.3 (4)	C17—C21—H21	118.2
C4—C3—H3	118.4	N7—C22—C23	110.5 (4)
C2—C3—H3	118.4	N7—C22—H22A	109.6
C3—C4—C5	117.4 (4)	C23—C22—H22A	109.6
C3—C4—C8	121.4 (5)	N7—C22—H22B	109.6
C5—C4—C8	121.2 (5)	C23—C22—H22B	109.6
C4—C5—C6	123.7 (4)	H22A—C22—H22B	108.1
C4—C5—H5	118.1	C22—C23—C24	112.6 (5)
C6—C5—H5	118.1	C22—C23—H23A	109.1
C5—C6—C1	117.3 (4)	C24—C23—H23A	109.1
C5—C6—C27	117.1 (5)	C22—C23—H23B	109.1
C1—C6—C27	125.6 (4)	C24—C23—H23B	109.1
C2—C7—H7A	109.5	H23A—C23—H23B	107.8
C2—C7—H7B	109.5	C23—C24—C25	110.1 (5)
H7A—C7—H7B	109.5	C23—C24—H24A	109.6
C2—C7—H7C	109.5	C25—C24—H24A	109.6
H7A—C7—H7C	109.5	C23—C24—H24B	109.6
H7B—C7—H7C	109.5	C25—C24—H24B	109.6
C4—C8—H8A	109.5	H24A—C24—H24B	108.1
C4—C8—H8B	109.5	C26—C25—C24	111.6 (5)
H8A—C8—H8B	109.5	C26—C25—H25A	109.3
C4—C8—H8C	109.5	C24—C25—H25A	109.3
H8A—C8—H8C	109.5	C26—C25—H25B	109.3
H8B—C8—H8C	109.5	C24—C25—H25B	109.3
C10—C9—C14	117.9 (3)	H25A—C25—H25B	108.0
C10—C9—N1	120.1 (3)	N7—C26—C25	110.3 (4)
C14—C9—N1	121.9 (3)	N7—C26—H26A	109.6
C11—C10—C9	122.3 (4)	C25—C26—H26A	109.6
C11—C10—H10	118.9	N7—C26—H26B	109.6
C9—C10—H10	118.9	C25—C26—H26B	109.6
C10—C11—C12	119.7 (4)	H26A—C26—H26B	108.1
C10—C11—H11	120.1	C6—C27—H27A	109.5
C12—C11—H11	120.1	C6—C27—H27B	109.5
O3—C12—C13	116.6 (3)	H27A—C27—H27B	109.5
O3—C12—C11	124.8 (4)	C6—C27—H27C	109.5
C13—C12—C11	118.6 (4)	H27A—C27—H27C	109.5
C12—C13—C14	121.7 (3)	H27B—C27—H27C	109.5
			
O1—S1—N1—C9	42.9 (3)	C12—C13—C14—C9	2.6 (6)
O2—S1—N1—C9	169.4 (3)	C12—C13—C14—S2	179.2 (3)
C1—S1—N1—C9	−74.5 (3)	C10—C9—C14—C13	1.8 (6)
O1—S1—C1—C2	174.6 (3)	N1—C9—C14—C13	177.9 (4)
O2—S1—C1—C2	44.2 (3)	C10—C9—C14—S2	−174.7 (4)
N1—S1—C1—C2	−68.3 (3)	N1—C9—C14—S2	1.4 (5)
O1—S1—C1—C6	−7.1 (4)	C16—S2—C14—C13	67.1 (3)
O2—S1—C1—C6	−137.5 (3)	C16—S2—C14—C9	−116.3 (3)
N1—S1—C1—C6	110.0 (3)	C18—N5—C16—C17	0.4 (5)
C6—C1—C2—C3	−2.4 (5)	C18—N5—C16—S2	−178.6 (2)
S1—C1—C2—C3	175.9 (3)	C14—S2—C16—N5	8.3 (3)
C6—C1—C2—C7	177.6 (4)	C14—S2—C16—C17	−170.8 (3)
S1—C1—C2—C7	−4.1 (5)	N5—C16—C17—C21	−2.3 (5)
C1—C2—C3—C4	1.0 (6)	S2—C16—C17—C21	176.7 (3)
C7—C2—C3—C4	−179.0 (4)	N5—C16—C17—Br1	178.8 (2)
C2—C3—C4—C5	0.8 (6)	S2—C16—C17—Br1	−2.2 (4)
C2—C3—C4—C8	−178.9 (4)	C26—N7—C18—N4	175.9 (4)
C3—C4—C5—C6	−1.3 (6)	C22—N7—C18—N4	5.7 (6)
C8—C4—C5—C6	178.4 (4)	C26—N7—C18—N5	−3.1 (6)
C4—C5—C6—C1	−0.1 (6)	C22—N7—C18—N5	−173.3 (4)
C4—C5—C6—C27	178.7 (4)	C21—N4—C18—N7	177.3 (4)
C2—C1—C6—C5	2.0 (6)	C21—N4—C18—N5	−3.7 (6)
S1—C1—C6—C5	−176.3 (3)	C16—N5—C18—N7	−178.3 (3)
C2—C1—C6—C27	−176.6 (4)	C16—N5—C18—N4	2.8 (5)
S1—C1—C6—C27	5.1 (6)	C18—N4—C21—C17	1.5 (6)
S1—N1—C9—C10	−71.3 (5)	C16—C17—C21—N4	1.3 (6)
S1—N1—C9—C14	112.7 (4)	Br1—C17—C21—N4	−179.8 (3)
C14—C9—C10—C11	−4.0 (8)	C18—N7—C22—C23	114.5 (5)
N1—C9—C10—C11	179.8 (5)	C26—N7—C22—C23	−56.6 (6)
C9—C10—C11—C12	1.7 (9)	N7—C22—C23—C24	53.4 (7)
C15—O3—C12—C13	−178.0 (4)	C22—C23—C24—C25	−52.3 (7)
C15—O3—C12—C11	5.4 (7)	C23—C24—C25—C26	53.1 (7)
C10—C11—C12—O3	179.3 (5)	C18—N7—C26—C25	−113.2 (5)
C10—C11—C12—C13	2.8 (8)	C22—N7—C26—C25	57.9 (6)
O3—C12—C13—C14	178.3 (3)	C24—C25—C26—N7	−55.7 (7)
C11—C12—C13—C14	−5.0 (6)		

### Hydrogen-bond geometry (Å, º)

**Table:**

*D*—H···*A*	*D*—H	H···*A*	*D*···*A*	*D*—H···*A*
N1—H1···O2^i^	0.86	2.03	2.880 (5)	172
C8—H8*A*···O2^ii^	0.96	2.48	3.242 (5)	136
C11—H11···O1^iii^	0.93	2.50	3.387 (6)	159

Symmetry codes: (i) −*x*, −*y*+2, −*z*; (ii) −*x*+1, −*y*+2, −*z*; (iii) −*x*, −*y*+1, −*z*.
